# Smartphone Applications for Mindfulness Interventions with Suicidality in Asian Older Adults: A Literature Review

**DOI:** 10.3390/ijerph15122810

**Published:** 2018-12-10

**Authors:** Carol C. Choo, Jonathan H. L. Kuek, André A. D. Burton

**Affiliations:** 1 College of Healthcare Sciences, James Cook University, Singapore 387380, Singapore; hkue4882@uni.sydney.edu.au; 2 School of Psychology, Curtin University, Perth 6102, Australia; andre.burton@postgrad.curtin.edu.au

**Keywords:** suicidality, Asian older adults, smartphone applications, mindfulness

## Abstract

Elderly suicide is a rising concern. Despite the advent of mobile technology, there remained a gap in the evidence base as to whether smartphone applications could be used for mindfulness intervention for suicidality in Asian older adults. This paper aimed to review recent research relevant to smartphone applications that could be used in providing mindfulness interventions for suicidality to Asian older adults. The inclusion criteria for this review were papers published in peer-reviewed journals from 2008 to 2018 with the usage of specific search terms, namely, ‘smartphone application’, ‘mobile application’, and ‘mindfulness’, assessed against the inclusion criteria and screened by an experienced Asian clinician to be of clinical utility for mindfulness intervention for suicidality with Asian older adults. Initial search on databases yielded 236 results. A total of 35 full text papers that fit the inclusion criteria were assessed for eligibility and 10 papers were included in the current review. This review highlighted the paucity of rigorous empirically validated research into effective smartphone applications that can be used for mindfulness interventions for suicidality with Asian older adults.

## 1. Introduction

Suicide rates increase with advancing age [1,2]. After the age of 75, suicide rates rise for both sexes for many Western countries [3]. In most Asian countries, suicide rates increase with age in both males and females but with a smaller male preponderance compared to their Western counterparts [1,4,5,6,7]. In both Taiwan and Japan, suicide rates recorded two peaks at the 20 to 30 years age group and the over 50 years age group [1]. The bimodal trend was recorded in earlier studies done in Hong Kong and Singapore [8]. Cheng and Lee [1] speculated that these ages coincided with the early and late onset for major depression. The strongest predictor of suicide in older adults was depression with comorbid alcohol intoxication [9].

Significant factors that contribute to suicide risk in older adults include: Age, psychiatric morbidity, chronic psychopathology, alcohol and drug dependence, cumulative losses, the breakdown of cardinal relationships, as well as subjective experience of loneliness and isolation [10,11]. Suicide risk assessment in older adults should consider stresses and strains related to multiple cumulative factors, including retirement, loss of work roles and income, living alone, social isolation, mental and physical illness, and low self-esteem [12]. Suicide notes from suicide deaths in Singapore revealed that the major reasons for suicide included physical illnesses in older adults [13]. Most suicides in older adults [13] suffered from depressive illness associated with debilitating physical illness, interpersonal problems, financial problems, or pre-existing social isolation or drug addiction. For these cases, depression was characterized by depressive moods, suicidal ideas, psychosomatic symptoms, and insomnia. 

In view of the rising suicide trends for older adults in Asia, the lack of convincing evidence on suicide treatment remains concerning. Although many studies have tried to assess the effectiveness of psychosocial or pharmacological treatments in reducing suicide attempts [14], overall, the results have been disappointing [15]. Recent advances in technology have ushered in an era for new developments in the delivery of mental health interventions [16]. Smartphone applications have shown potential in reducing the healthcare cost for treating psychiatric illnesses in Asia [17,18]. In comparison to Western countries, there is a shortage of mental health professionals in Asia, yet a high proliferation of mobile phone usage throughout Asia [19]. Over 50% of the Asian population use smartphones, with Singapore alone reporting that the smartphone adoption rates far exceed the population [20].

The evidence base for use of smartphone applications has been demonstrated in many areas [21,22,23,24,25,26,27,28,29,30,31], and internet-based interventions have been found to be efficacious for mental health issues [32] strengthening support [33], overall motivation [34], enhancing coping, and facilitating recovery [35,36] and with ethnically diverse populations [37]. In view of the recent advances in technology, this holds promise for mental health professionals to develop smartphone applications as an alternative platform to deliver interventions [38]. 

Some clinics in Australia have implemented conjunctive treatment modalities in programs such as cognitive behavioral therapy and psychoeducation applications alongside face-to-face therapy [37]. One example is the dialectical behavioral therapy coach [39]. This application aims at cultivating emotional regulation skill and changing negative emotions [40]. Such developments are currently lacking in Asia. It could not be assumed that smartphone applications delivering effective interventions in Western cultures would be similarly effective in Asian cultures [41]. Cultural adaptations might be needed. As age contributes to suicide precipitants [42], more research would be needed for usage in the elderly. Culture plays a pivotal role in determining risk and protective factors for suicidality, which informs targeted intervention strategies [41]. However, the evidence base for suicide interventions using smartphone applications seems largely unexplored in Asian older adults.

The evidence base for mindfulness interventions in Asia has gained momentum within the current decade [43]. Mindfulness interventions have been used to treat various psychological problems, such as depression [43,44,45]. Depression is a common psychiatric illness in Asia. Asians suffering from depression often experience maladaptive ruminations [46] and would be suitable for mindfulness-based therapy [47]. Furthermore, older adults vulnerable to suicidality are often affected by various issues, such as debilitating chronic diseases [42]. Mindfulness-based therapy has shown evidence [48] that it can enhance resilience and reduce vulnerability Asians with chronic diseases [49]. Recent studies have highlighted the links between resilience, suicidality [41,50], and mindfulness practice in Asian populations [49,50]. In Asia, the stigma related to mental illness and suicidality might hinder help-seeking behavior [51]. These vulnerable older adults might prefer to access self-help instead [19], and smartphone applications could offer a cost-effective [52] alternative self-help platform. The accessibility of such applications could enhance our efforts in primary prevention and mental health promotion. A recent study in Singapore highlighted the need for mental health promotion to reduce stigma related to psychiatric illness and enhance psychological wellbeing [49]. Recent research indicated that preventative mental healthcare involves enhancing resilience, which includes the use of mindfulness-based interventions for emotional regulation [41,46,49]. However, the acceptability and perspectives of Asian older adults remains unclear. There was an indication that older age might relate with less time of usage of a novel mindfulness smartphone application in Spanish adults [52], but the phenomenon among older adults in Asia remains largely unexplored. User perspectives would be important, and the ease of use might influence continued usage [53]. In view of the aforementioned literature review, a gap continues to exist for an evidence base for mindfulness-based suicide interventions using smartphone applications in Asian older adults.

There were many smartphone applications currently available that are marketed as mindfulness applications. Using the search term “mindfulness-based iPhone Applications” from November 2013 yielded 808 results. This number was consistent with earlier research informed by a search for “mindfulness” conducted on iTunes and Google Applications for mindfulness training [33]. Such applications were reviewed by experts. However, the utility among Asian older adult consumers remains unclear. Widespread implementation of self-help mindfulness interventions could be premature without concrete evidence and scientific scrutiny for use by the intended population [54]. Rigorous scientific enquiry should be applied to explore the therapeutic benefits [55] of such applications for older adults in Asia. Research aimed at examining low-cost smartphone applications that could be efficacious as a therapeutic tool for suicidality in Asian older adults would add significantly to the current literature [56]. Considering the need for early prevention in suicidality [49], research is much needed to explore alternative ways to deliver effective interventions for older adults, which are also cost effective and easily accessible. The aim of this paper is to review research relating to the evidence base for smartphone applications that can be used for mindfulness intervention for suicidality in Asian older adults.

## 2. Methods

The inclusion criteria for this review were publications in peer-reviewed journals from 2008 to 2018, with the usage of specific search terms, namely, ‘smartphone application’, ‘mobile application’, and ‘mindfulness’. The databases examined included PSYCINFO, SCOPUS, Google Scholar, Medline, and PubMed. The papers were retrieved if they related to interventions delivered using smartphone applications for mindfulness interventions. The structured proforma for evaluating eligibility for inclusion involved the following: Recent papers that contained original work published in peer-reviewed journals after the year 2008; related to the usage of smartphone applications by clinicians for therapeutic purposes and considered by an experienced Asian clinician to be of clinical utility with suicidal older adults in Asia. The main purpose was to obtain primary citations on studies which were completed, and not review papers that fit the inclusion criteria. The reason for exclusion were articles that did not refer to the use of smartphone applications by clinicians for therapeutic purposes. 

## 3. Results

**Figure 1:**
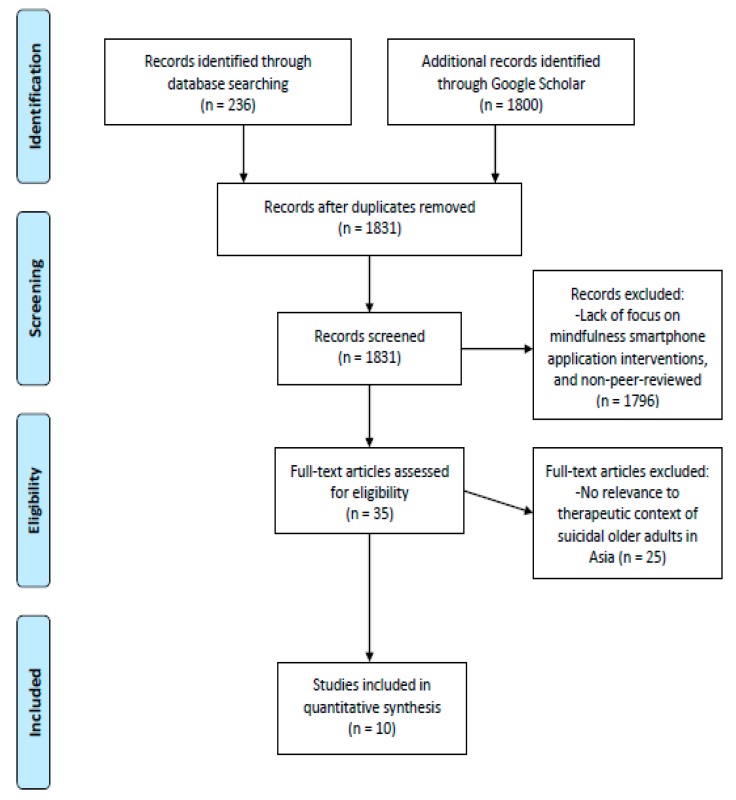
PRISMA flow diagram.

The aforementioned databases were initially used to identify peer-reviewed papers with the inclusion criteria named above, which yielded 236 results, using all search terms. Additional records were identified through Google Scholar and yielded 1800 additional results. From the original search results, 205 duplicated articles were removed, and 1831 abstracts were screened; 35 full text papers from peer-reviewed journals were then downloaded and assessed against the inclusion criteria. Papers were excluded mainly due to these reasons: They were not original research published in peer-reviewed journals after the year 2008, or they were not related to usage of smartphone applications by clinicians for therapeutic purposes with clinical utility for suicidal older adults in Asia. Review papers were excluded. See Figure 1 for the PRISMA flow chart [57]. The results of the review are presented in Table 1. Ten recent papers deemed to be suitable were included in the current review.

A review of papers presented in Table 1 demonstrated the lack of convincing evidence for the efficacy of mindfulness interventions delivered via smartphone applications that could be used for suicide interventions for Asian older adults. These papers were examined by extracting pertinent information, namely, the study design, sample characteristics, primary objective, and outcome measures, and if these results were significant, this pertinent information was summarized (Table 1). Only quantitative studies were included in the formal review and are presented in Table 1. Two qualitative papers [57,58] were also considered but not included in the formal review in Table 1. The first four papers presented in the table were nonrandomized controlled trials, while the rest of the six studies were randomized controlled trials. A longitudinal study [43] and randomized controlled trials [30] showed that mindfulness interventions delivered using smartphone applications significantly improved the psychological, social, and environment quality of life [30] and the levels of mindfulness, significantly reduced depression [28,29] and general psychiatric symptoms [30], and worked better for those with lower initial severity on depression [29]. Inconsistent results were found for the outcome of stress. No significant result was found in randomized controlled trials done in two studies [27,59] on stress, but a randomized controlled trial on students [31] found a significant decrease in perceived stress and increase in wellbeing [31]. Other studies examined outcomes on user attitudes and usage data [51,60], which were not related to the focus on suicidality. None of these studies included any outcome measures of suicidality except Ly et al. [29], which used PHQ-9 and BDI-II. The studies reviewed included participants whereby mean ages ranged from 25.11 to 45.63, which were not representative of older adults. All the studies reviewed had limited or no representation of Asians at all. In summary, the extent of generalizability of such findings to suicidality in Asian older adults remains questionable. The quantitative studies reviewed in Table 1 indicated that considerations for future research should include intervention lasting more than 10 days, with more than one post-intervention measurement [28], and include personalized experience [52]. Barriers such as negative perceptions and negative emotions [58] might affect continued usage.

## 4. Discussion

In view of the review of recent papers presented in Table 1, although six of the ten studies employed a robust methodology of randomized controlled trials, a gap remains in convincing evidence for the efficacy of mindfulness interventions delivered via smartphone applications that could be used for suicide interventions for Asian older adults. It is concerning that there was a lack of interactive self-care applications available to Asian older adult consumers incorporating initial screening for suitability or offering targeted guidance regarding the management of suicidal crisis [61]. Few of the applications currently in the market included content aimed at encouraging professional help-seeking or had an explicit description of the theoretical or empirical basis of interventions. This gap could be addressed by partnerships between clinicians with software engineers and specialists in biomedical informatics to develop, test, and refine appropriate applications. When designing such an application, features could include: An evidence base supporting the use of mindfulness techniques in Asian older adults, as well as older adults’ user perspectives. The initial phase of the development of such applications should also consider a pilot study to explore older users’ perspectives, such as the ease of use [52,57]. Videos and information could be incorporated [51], as well as music [27] and relaxation skills [52]. 

Mindfulness features in the application could include: Breathing, body scanning, sitting meditations, loving kindness meditations, thoughts and emotion focus, mountain meditation, lake meditation, and three-minute breathing spaces [45]. It is noteworthy that the specific aspects of mindfulness activities which could improve suicide outcomes in Asian older adults had not been explored and should be included in future studies with Asian older adults. The content of applications for suicidality should also contain at least one interactive suicide prevention feature, e.g., safety planning, facilitating access to local crisis support, and contain at least one strategy consistent with the evidence base or relevant best-practice guidelines [62]. Psychoeducational components to reduce the stigma related to suicidality and mental illness could be incorporated [40], together with the monitoring of moods and stressors or other suicide triggers [48]. Older adults were often adversely affected by many psychosocial stressors, such as debilitating chronic illnesses. These stressors should be carefully addressed [50], and multidisciplinary interventions could be indicated to adequately address various aspects of chronic health issues, in view of the close links between mental wellbeing and physical wellbeing [41].

Another consideration is that suicidal Asian older adults were not a homogenous group [50]. Suicide risk assessment should be conducted with a consideration of suicide risk and protective factors [48] in older adults, e.g., alcohol and drug dependence, losses, relationship breakdowns, and isolation [10,11,12], which could be carefully screened. When these factors are detected, referrals should be promptly made to relevant professionals, e.g., to assess and manage severe depressive illness, debilitating physical illness, and to gain help with financial problems or pre-existing social isolation or drug addiction. 

Therapeutic needs must be considered before clinicians decide on the suitability for use of a mindfulness application with their patients. Clinicians should carefully examine the prevailing code of ethics in working with suicidal clients to ensure best practice is observed [40,50]. This might include a comprehensive suicide risk assessment before deciding on the best intervention for the client [44]. Another factor to consider is to define the primary therapeutic goal and outcome, e.g., reduced intensity or frequency of suicidal ideation [40,50], and monitor the therapeutic gains progressively. It remained unclear if suicide risk screening and monitoring using a smartphone application could replace face-to-face assessment conducted by an experienced clinician, but it would seem that the prevailing code of ethics and professional best practice currently would not support this [40,48,50], especially when the evidence base has not been clearly demonstrated. 

Future research could focus on empirical and randomized controlled trials with older Asian adult samples that conform to CONSORT guidelines [63], with the inclusion of standardized suicidality outcome measures, e.g., PHQ-9 and BDI-II. Nevertheless, the strength of the review included the investigation of an important clinical issue, highlighted promising results mainly in Western adult samples, but underscored the need for more research on this pertinent topic for Asian older adults.

## 5. Conclusions

In summary, suicidal risk in older adults is a rising concern, globally and in Asia [50]. The potential use of smartphone applications in the delivery of mindfulness intervention tailored for suicidality in Asian older adults remains promising, but an evidence base to support its use is currently lacking. More research is needed to address the current gaps in knowledge and to provide a rigorous evidence base for the implementation of smartphone technologies in older adults. Developing mobile tools for suicidal older adult users requires careful ethical consideration regarding the patient–practitioner relationship, user perspectives, acceptability, ease of use, the logic of self-surveillance, the prevailing code of ethics, and overall best practice. More rigorous research and evaluations are needed to ascertain the efficacy and establish evidence for best practice for the usage of such smartphone applications [37].

## Figures and Tables

**Table 1:** Summary of Evidence.

Author/Year	Study Design/Participants Characteristics/Grouping	Study Objectives	Results
Plaza Garcia, Sanchez, Espilez, Magarino, Guillen, and Garcia-Campayo (2017) [51]	Nonrandomized controlled trial 3977 users were involved in this study: 26 in the first trial during an 8-week usage period and 3951 in the second trial for 17 months (7.7% of the sample were below the age of 29 years)	The aim of the study was to examine a first prototype of a smartphone application with Spanish features for the training and practice of mindfulness.	In the first study, participants assessed the application and considered it a helping tool for mindfulness practice, and user-friendly. In the second study, weak associations were found between usage time and age, nationality, and educational level. The mindfulness level showed a weak positive correlation with the session accomplished (*p* = 0.051). Videos and information stood out as the most accessed resources.
Carissoli, Villani, and Riva (2015) [27]	Controlled pragmatic trial 56 Italian workers (*M*_age_ = 38.11, *SD_age_* = 6.92) were block-randomized to three conditions (self-help mindfulness, relaxing music, and wait list)	The aim of this study was to examine the efficacy of a 3-week mindfulness-inspired protocol, delivered by an Android application for smartphones, in reducing stress in the adult population.	No significant differences between groups on any of the perceived stress dimensions measured (*p* = 0.103–0.952). Participants in the mindfulness group reported significant reductions in hyperactivity and accelerated behaviors (*p* = 0.010).
Chittaro and Vianello (2016a) [43]	Mixed methods, longitudinal study 3979 participants downloaded the app but only 136 (*M_age_* = 37.85, *SD_age_* = 11.30) completed all the questionnaires required. No grouping or specific demographical data.	The aim of this study was to assess the effectiveness of a mobile mindfulness app at increasing levels of mindfulness.	The results revealed that users with no or minimal experience with meditation significantly increased in their levels of mindfulness from the start and at the end of the 4-week study period (*p* < 0.001). Additionally, qualitative feedback indicated that the app was positively perceived as beautiful and its usage elicited positive feelings in the participants.
Donovan et al. (2016) [52]	Mixed methods, nonrandomized controlled trial 20 participants (*M_age_* = 16.9, *SD_age_* = 1.3) of ethnically diverse origins (7 African American, 3 Black Latino, 2 Hispanic, 2 Asian, 1 Native American, 1 Latino, and 4 Caucasian).	The aim of the study was to test the feasibility of a mindfulness and self-compassion based program for adolescents, delivered though mobile phones over a 30-day period.	Outcome measures included usage, satisfaction, and focus group findings. Usage data showed that participants used the app on the majority of the 30 days (*M* = 16.8, *SD* = 6.4, *Range* = 6–26). Satisfaction data indicated that they enjoyed using the program (92%), found it easy to navigate (72%) and understand (86%), and that the program helped them to focus on their feelings (71%) and learn new relaxation skills (64%). A total of 64% indicated they would be likely to continue using it. Qualitative data corroborated these findings but also provided suggestions for improvement (e.g., a more personalized experience).
Economides, Martman, Bell, and Sanderson (2018) [59]	Randomized controlled trial 69 participants randomized into headspace (*n* = 41, 75.6% White, 4.9% Hispanic, 2.4% African American, 9.8% Asian, 4.9% Mixed, and 2.4% others) and audiobook conditions (*n* = 28, 64.3% White, 3.6% Hispanic, 17.9% Asian, 7.14% Mixed, and 7.14% others) completed the study.	The study aimed to assess whether completing the first 10 introductory sessions of the mindfulness-based smartphone app Headspace positively impacted stress, affect, and irritability, relative to an active control (audiobook on mindfulness, which differed only on content).	The results suggested that there was no significant difference between both interventions, as they were equally effective at reducing stress associated with personal vulnerability (*p* = 0.09, *d* = 0.26); but only the mindfulness intervention had a significant positive impact on irritability (*p* < 0.05, *d* = 0.44), affect (*p* < 0.001, *d* = 0.47), and stress resulting from external pressure(*p* < 0.001, *d* = 0.45).
Hoswells, Iytzan, and Eiroa-Orosa (2016) [28]	Randomized controlled trial 121 participants (*M_age_* = 40.7, *SD_age_* = 10.6) were assigned to the experimental (57) and control condition (64). The sample consisted pf 90.1% Caucasian, 1.7% Asian/Pacific Islander, 1.7% Hispanic, 5.0% other/multiracial, and 1.5% who declined to respond.	The objective of the study was to measure the efficacy of a mindfulness-based smartphone application designed to enhance wellbeing.	The findings on the mindfulness meditation application Headspace found that it was associated with a significantly increased positive affect (*p* = 0.003) and decreased depression (*p* = 0.05). No statistically significant difference in satisfaction with life or negative affect was found and might be attributable to the limited time duration of the research (intervention only lasted for 10 days) and there was only one post-intervention measurement.
Ly et al. (2014) [29]	Randomized controlled trial 81 participants (*M_age_* = 36.0, *SD_age_* = 10.8) diagnosed with major depressive disorder were randomized into a behavioral activation treatment (*n* = 40) and mindfulness treatment (*n* = 41). No specific demographic information.	The aim of the study was to test the effects of two smartphone-delivered treatments, and to find out if the behavioral activation treatment was more effective than the mindfulness treatment. To evaluate the long-term effects, a 6-month follow-up after the start of the treatment was also included.	The results showed no significant interaction effect of group and time on any of the outcome measures either from pretreatment to post-treatment or from pretreatment to the 6-month follow-up. Subgroup analyses showed that the behavioral activation treatment was more effective than the mindfulness treatment among participants with higher initial severity of depression from pretreatment to the 6-month follow-up based on the PHQ-9 (*p* < 0.05, *d* = 0.47). Mindfulness treatment worked better among participants with lower initial severity from pretreatment to the 6-month follow-up based on the PHQ-9 (*p* < 0.01, *d* = 0.98) and BDI-II (*p* < 0.05, *d* = 1.21).
Van Emmerik, Berings, and Lancee (2018) [30]	Randomized controlled trial 377 participants were randomized into either a mindfulness (*n* = 191, *M_age_* = 45.63, *SD_age_* = 9.09, 4.2% male, 95.8% female) or waitlist control condition (*n* = 186, *M_age_* = 43.78, *SD_age_* = 10.48, 3.8% male, 96.2% female)	The aim of the study was to evaluate the immediate and long-term efficacy of an MBI app (the VGZ Mindfulness Coach) in a wait-list controlled randomized trial, with mindfulness as a primary outcome variable and without any form of therapeutic guidance in addition to the self-help app.	The results revealed that compared to the waitlist control, the mindfulness intervention group, large (Cohen’s d = 0.77) and statistically significant increases of mindfulness after 8 weeks and small-to-medium increases of the Observing, Describing, Acting with awareness, Nonjudging, and Nonreactivity mindfulness facets as measured with the five facet mindfulness questionnaire (Cohen’s *d* = 0.66, 0.26, 0.49, 0.34, and 0.43, respectively) were reported. There were also large decreases of general psychiatric symptoms (GHQ-12; Cohen’s *d* = −0.68) and moderate increases of psychological, social, and environmental quality of life (WHOQOL-BREF; Cohen’s *d* = 0.38, 0.38, and 0.36, respectively). Except for social quality of life, these gains were maintained for at least 3 months.
Wolf, Kraft, Tschauner, Bauer, Becker, and Puschner, (2016) [60]	Randomized controlled trial 41 patients were randomized into either a text message mindfulness intervention (*n* = 21, *Mage* = 43.38, *SDage* = 12.65) or control group (*n* = 20, information about control group not provided). No other demographic data.	This study investigated the user activity in a text messaging intervention to assist mindfulness practice in patients with symptoms of depression.	The results revealed that women sent more messages than men (*d* = 0.73), and age was moderately correlated with the number of messages sent (*r* = 0.39). Prior text messaging experience was negatively associated with the activity of participants as more experienced participants sent fewer messages (*r* = −0.42). Participants who attended more mindfulness group sessions were also sending more messages (*r* = 0.40). Prior mindfulness experience also led to more messages being sent (*d* = 0.25). The number of messages sent was correlated with reductions in depressive symptoms (*r* = −0.46), preservative thinking (*r* = −0.36), and moderately associated with increases in self compassion(*r* = 0.45) and mindfulness (*r* = 0.23).
Yang, Schamber, Meyer, and Gold (2018) [31]	Randomized controlled trial 8 medical students (*M_age_* = 25.11, *Range* 21–47; 63.6% female, 36.4% male; 25% Asian/Pacific Islander, 6.8% Black, 46.6% Caucasian, 5.7% Latino, 10.2% Mixed, and 5.7% others) were stratified by class year and randomized to either mindfulness intervention (*n* = 45) or control group (*n* = 43).	This study assessed whether 10–20 min of daily mindfulness meditation for 30 days, using a mobile phone application, could decrease perceived stress and improve wellbeing for medical students.	All participants completed the perceived stress scale (PSS), five-facet mindfulness questionnaire (FFMQ), and general wellbeing schedule (GWBS) at baseline, 30 days, and 60 days. There was a significant interaction between time and treatment group for perceived stress and wellbeing. Perceived stress significantly decreased for the intervention group (*p* < 0.05). General wellbeing significantly increased for the intervention group compared to the control group and the increase was sustained (*p* < 0.05).
